# Sudden bilateral vision loss due to third ventricular cavernous angioma with intratumoral hemorrhage - case report

**DOI:** 10.1186/s12886-019-1252-5

**Published:** 2019-12-16

**Authors:** Kan Ishijima, Yasuhiro Shinmei, Mayo Nozaki, Shigeru Yamaguchi, Shinki Chin, Susumu Ishida

**Affiliations:** 1 Department of Ophthalmology, Hokkaido University Graduate School of Medicine, North 15, West 7, Kita-ku, Sapporo, 060-8638 Japan; 2 Umekita Eye Clinic, Osaka, Japan; 3 Department of Neurosurgery, Faculty of Hokkaido University Graduate School of Medicine, Sapporo, Japan

**Keywords:** Sudden bilateral vision loss, Third ventricular cavernous angioma, Intratumoral hemorrhage, Chiasmal syndrome, Contrast-enhanced MRI

## Abstract

**Background:**

We report a rare case of sudden bilateral vision loss due to third ventricular cavernous angioma with intratumoral hemorrhage.

**Case presentation:**

A 45-year-old woman presented decreased visual acuity in both eyes. Her best corrected visual acuity was 0.1 in the right eye and 0.15 in the left eye. Goldmann perimetry showed bilateral central scotomas and bitemporal visual field defects. MRI demonstrated a lesion with mixed hypo- and hyperintensity at the optic chiasm, which was thought to be an intratumoral hemorrhage. The patient underwent bifrontal craniotomy. The tumor was exposed via an anterior interhemispheric approach, and histological evaluation of the mass led to a diagnosis of cavernous angioma. Six months after the surgery, her best corrected visual acuity was 0.9 in the right eye and 0.9 in the left, with slight bitemporal visual field defects.

**Conclusion:**

Third ventricular cavernous angioma is considered in the differential diagnosis of chiasmal syndrome. Contrast-enhanced MRI and FDG-PET might be useful for differential diagnosis of cavernous angioma from other chiasmal tumors including glioblastoma**.**

## Background

Chiasmal syndrome is associated with lesions of the optic chiasm, manifesting as various impairments of the visual field such as central scotoma, bitemporal paracentral scotoma, junction scotoma and bitemporal hemianopsia [1]. Pituitary tumors are the most common cause [2], but it may be caused by neurofibromatosis, angioma and glioma [3], and associated with other inflammatory diseases such as hypophysitis, chiasmal neuritis, and multiple sclerosis [4].

We report a rare case of chiasmal syndrome with sudden bilateral vision loss due to third ventricular cavernous angioma presenting with intratumoral hemorrhage.

## Case presentation

A 45-year-old woman presented decreased visual acuity in both eyes for three days. She visited an ophthalmic clinic where she was diagnosed as having bilateral optic neuritis with central scotoma and was referred to our hospital. A week earlier, she had complained of headaches and visited a neurologist, but no abnormality was found on brain magnetic resonance imaging (MRI) and neurological tests. She had no history of ocular or systemic disease.

**Fig. 1:**
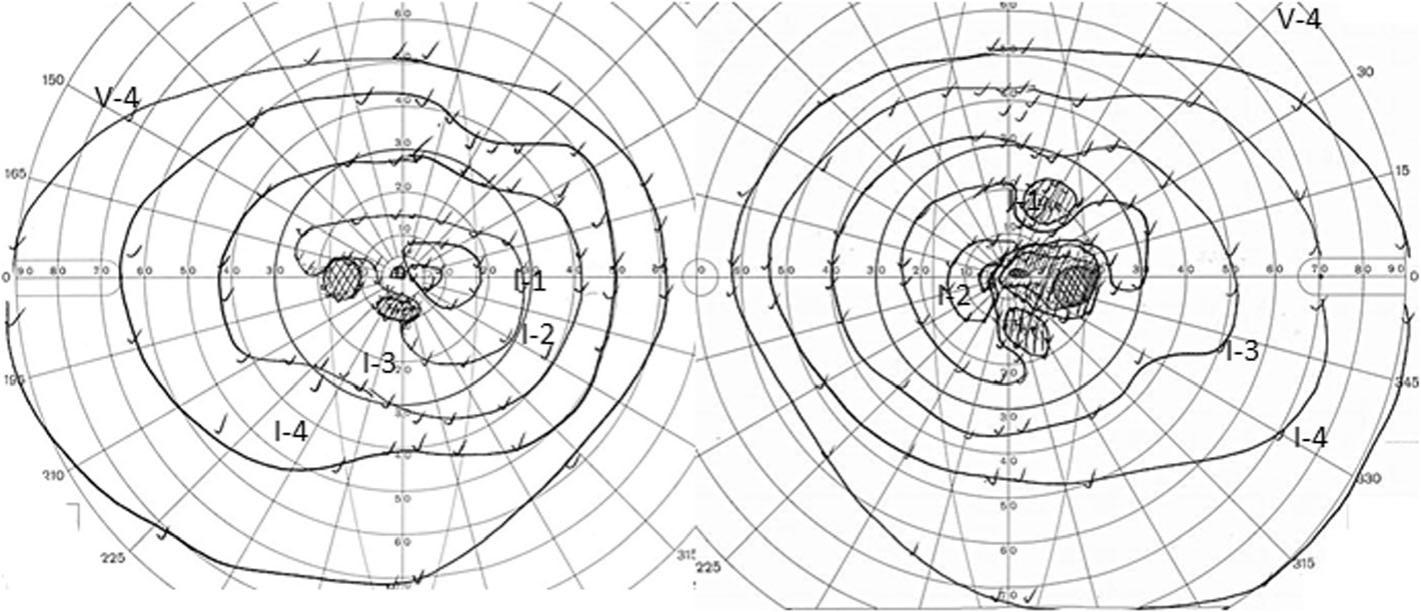
Goldmann perimetry showing bilateral central scotomas and bitemporal visual field defects

**Fig. 2:**
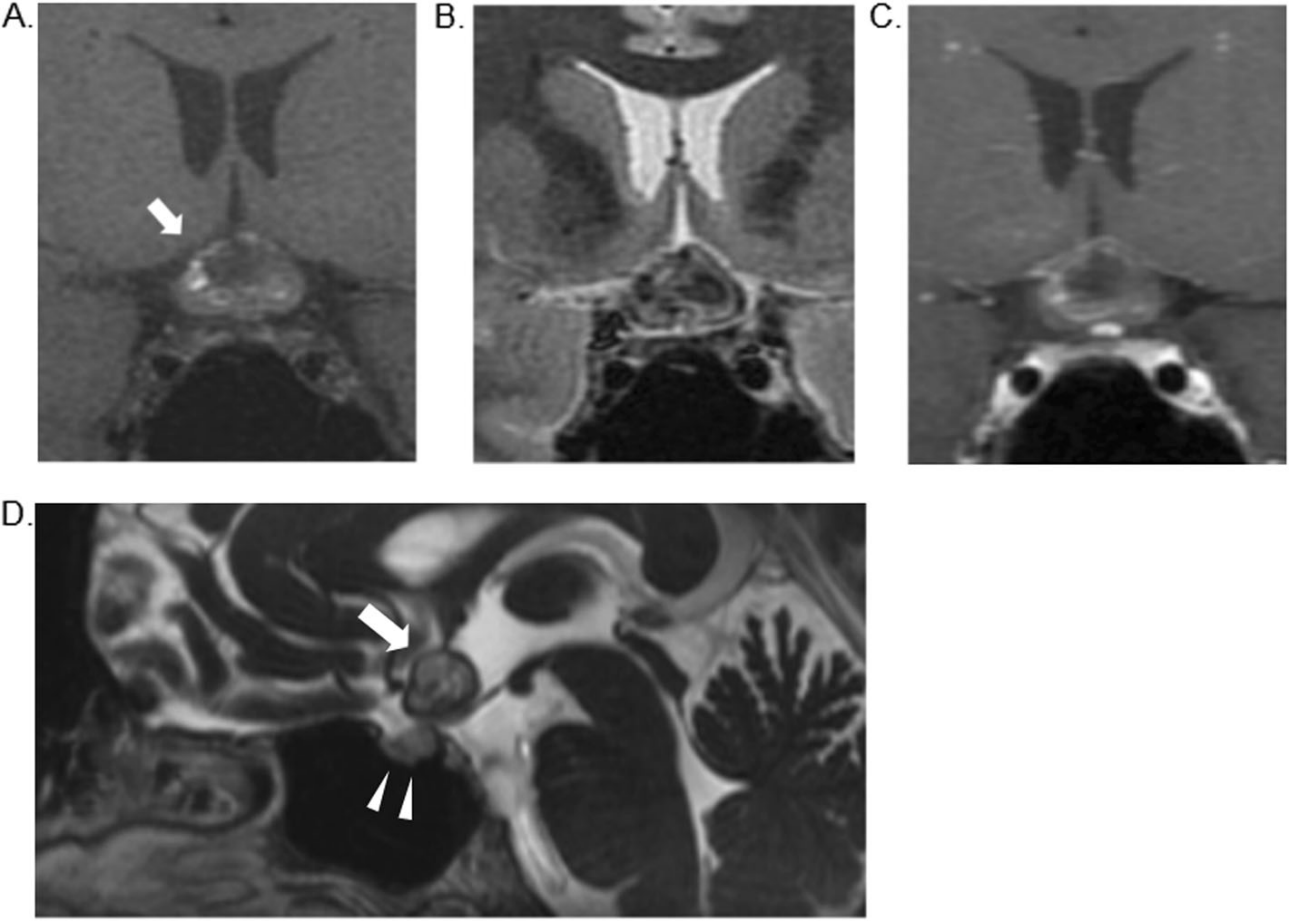
Coronal T1 weighted MRI showing a lesion with mixed hypo- and hyperintensity (white arrow) (**a**). Coronal T2 weighted MRI (**b**). Coronal post-gadolinium contrast-enhanced MRI showing minimal enhancement around the lesion (**c**). Sagittal MRI with contrast-enhanced constructive interference in steady state revealed the lesion (white arrow; major axis: 12.8 mm) (**d**). White arrow heads indicate the sella

Upon our examination, best corrected visual acuity was 0.1 in the right eye and 0.15 in the left eye. The pupillary light reflex was sluggish **i**n both eyes. Slit-lamp examination revealed no signs of ocular disease. Fundus examination was normal. Goldmann perimetry showed bilateral central scotomas and bitemporal visual field defects (Fig. 1). The pattern visual evoked potential showed prolonged P100 latency in both eyes; 138 msec in the right eye and 142 msec in the left. Contrast-enhanced MRI of the brain and orbits was performed again due to suspected optic neuritis. T1- and T2 weighted MRI showed a lesion with mixed hypo- and hyperintensity at the optic chiasm, which was thought to be an intratumoral hemorrhage (Figs. 2a, b). Contrast-enhanced MRI showed minimal enhancement around the lesion (Fig. 2c). Sagittal MRI with contrast-enhanced constructive interference in steady state showed the lesion in front of the third ventricle (Fig. 2d).

We diagnosed her as having bilateral compressive optic neuropathy due to chiasmal tumor and referred her to neurosurgeons for further examination and treatment. Fluorodeoxyglucose positron emission tomography (FDG-PET) performed for differential diagnostic purposes did not show an increased FDG accumulation in the tumor. Her visual acuity had spontaneously improved to 0.9 in the right eye and 0.8 in the left with a disappearance of bilateral central scotoma since her first visit to our clinic. Spinal fluid examination showed laboratory data within normal limits. Pituitary function test showed that TSH was high, at 13.0μIU/ml, but other values were within normal limits.

**Fig. 3:**
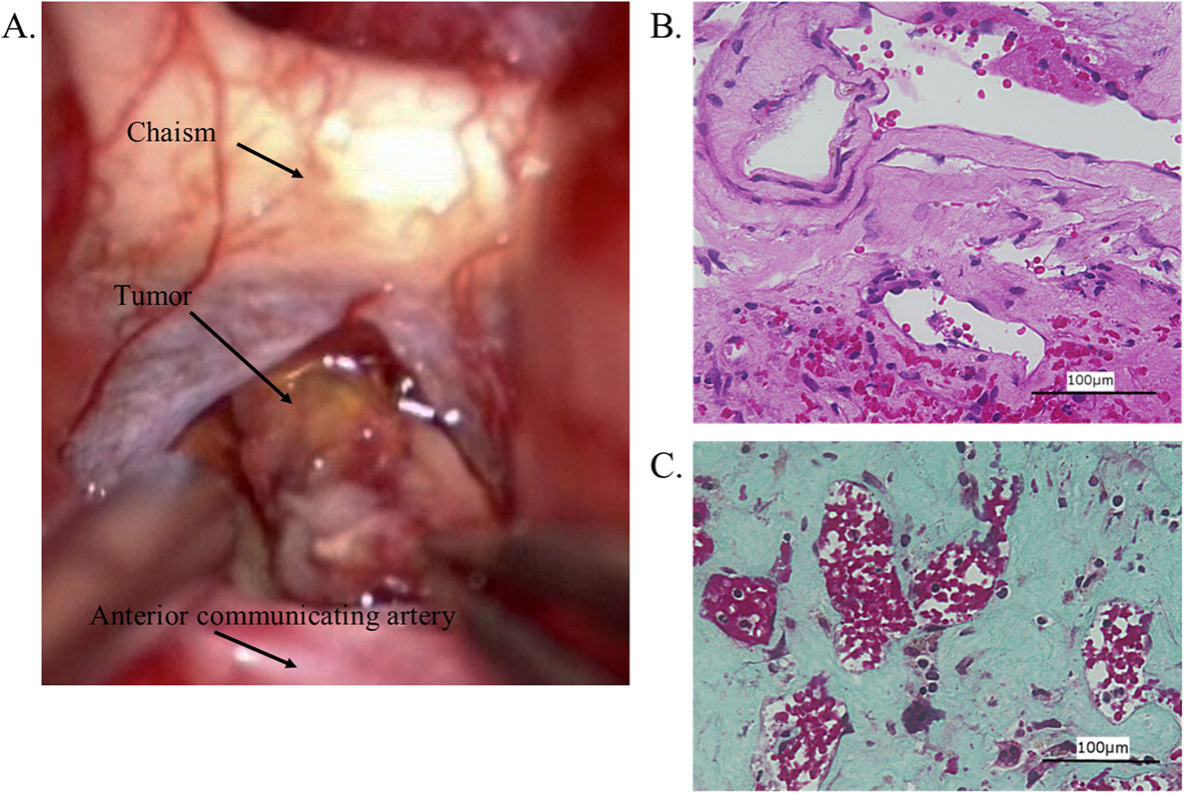
A yellow mass developed from the anterior wall of the third ventricle to the lamina terminalis on the chiasm, seen intraoperatively (**a**). HE staining shows many lumen structures with hemosiderin deposits, as well as infiltration of inflammatory cells (**b**). Elastica-Masson staining shows copious collagen fibers around the lumen structure (**c**)

Despite spontaneous improvement in visual acuity, the patient underwent bifrontal craniotomy to prevent rebleeding 48 days after onset, and the tumor was exposed via an anterior interhemispheric approach. A yellow mass, developed from the anterior wall of the third ventricle to the lamina terminalis on the chiasm, was seen intraoperatively (Fig. 3a). After the surgery, the tumor could be distinguished from the chiasm and was completely resected with preservation of the optic nerve. Histological evaluation of the mass led to a diagnosis of cavernous angioma. HE staining showed many lumen structures with hemosiderin deposits, as well as infiltration of inflammatory cells (Fig. 3b). Elastica-Masson staining revealed copious collagen fibers around the lumen structure (Fig. 3c).

**Fig. 4:**
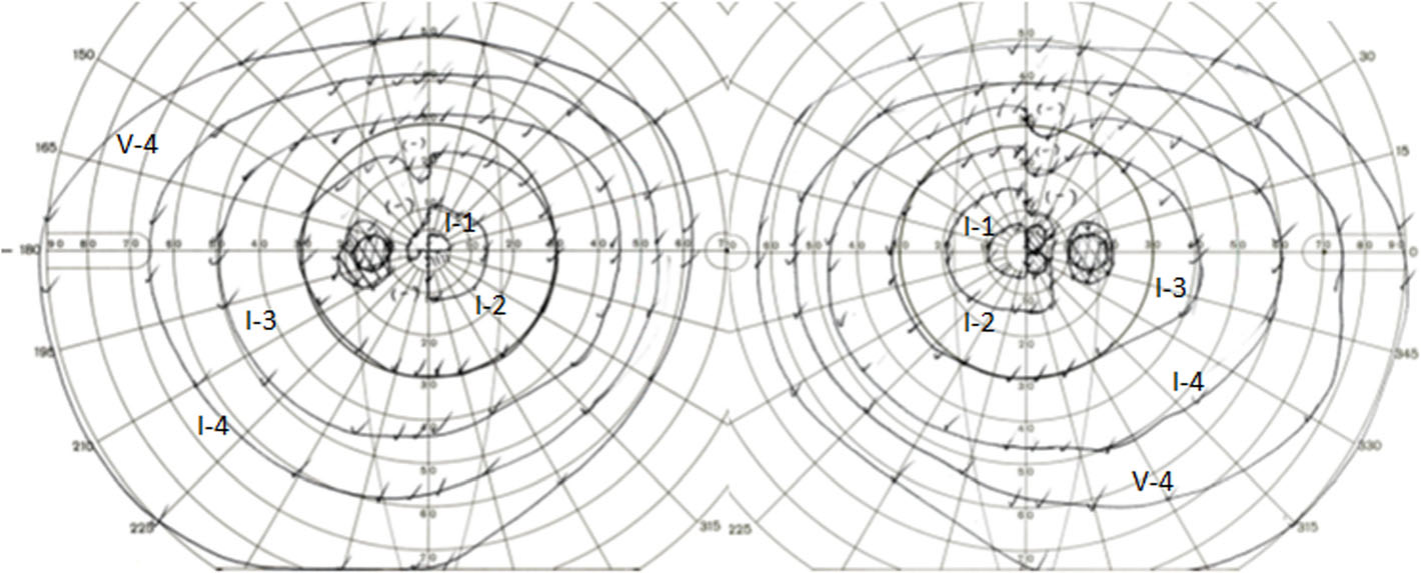
Goldmann perimetry at six months after surgery showing visual field recovery with slight bitemporal defects

Six months after the surgery, her best corrected visual acuity was 0.9 in the right eye and 0.9 in the left, with slight bitemporal visual field defects (Fig. 4).

## Discussion and conclusions

Cavernous angioma (CA) of the central nervous system accounts for 8 to 15% of cerebrovascular malformations in postmortem studies [5]. Third ventricle cavernous angioma is a most unusual variant of CA [6–8], with only 39 cases in the literature [9]. According to this review, these patients presented at a median age of 38 years and the most common symptom was headaches (26 patients; 66.7%). Of these patients, 24 were female (61.5%) and 15 were male (38.5%). In terms of specific location within the third ventricle, 18 patients (46.2%) had CA at the Foramen of Monro (FoM), 7 (17.9%) had CA in the lateral wall of the third ventricle, 3 (7.7%) had CA in the floor of the third ventricle, 3 (7.7%) had CA in the suprachiasmatic region, 5 (12.8%) had CA in the pineal region/posterior third ventricle, and 3 (7.7%) had CA at an unspecified location.

CAs located at the FoM were the most likely to cause hydrocephalus (83.3%) followed by CAs located at the pineal region/posterior third ventricle (80%). On the other hand, CAs located in the suprachiasmatic region were most likely to cause endocrine dysfunction (33%), because of the close to the hypothalamus. Visual disturbances were also seen in CAs located in the suprachiasmatic region (100%).

Our patient also developed headaches and presented increasing TSH. As mentioned, suprachiasmatic CAs sometimes cause endocrine dysfunction, because they are closed to hypothalamus which controls the pituitary gland. Further, the hemorrhage in the CA had compressed the chiasm and caused sudden bilateral vision loss in this case. The hemorrhage was absorbed, which may explain the spontaneous improvement in visual function before the surgery. A important issue with CAs is their potential to bleed [6–8]. CA may have an autosomal dominant inheritance, and not all lesions require surgery. However, surgery is advocated for those with severe vision loss or recurrent hemorrhage [10, 11].

In this present case, MRI detected mixed hypo- and hyperintensity at the chiasm, so the sudden vision loss of both eyes would have been cause by chiasmal compression due to acute intratumoral hemorrhage. Previous cases have shown that intratumoral hemorrhage is a common finding in glioblastoma [12]. In this case, contrast-enhanced MRI did not show abnormal enhancement of the tumor, and FDG-PET did not detect increased FDG accumulation at the tumor***.*** These findings are different from previous reports of malignant tumors including glioblastoma**.** Because of these preoperative imaging results and the spontaneous visual recovery before the operation, we supposed the lesion might be CA rather than glioblastoma. Although MRI could not distinguish the tumor from the chiasm, they were actually separable at the surgery and her visual function recovered further after the surgery.

Third ventricular CA is considered in the differential diagnosis of chiasmal syndrome. Contrast-enhanced MRI and FDG-PET might be useful for differential diagnosis of CA from other chiasmal tumors including glioblastoma**.**

## Data Availability

Not applicable.

## Abbreviations

CA: Cavernous angioma
FDG-PET: Fluorodeoxyglucose positron emission tomography
FoM: Foramen of Monro
MRI: Magnetic resonance imaging

## References

[1] Foroozan R (2003). Chiasmal syndromes. Curr Opin Ophthalmol.

[2] Molitch ME (2017). Diagnosis and treatment of pituitary adenomas: a review. JAMA..

[3] Farazdaghi MK, Katowitz WR, Avery RA (2019). Current treatment of optic nerve gliomas. Curr Opin Ophthalmol.

[4] Kawasaki A, Purvin VA (2009). Idiopathic chiasmal neuritis: clinical features and prognosis. Arch Ophthalmol.

[5] Zakaria MA, Abdullah JM, George JP (2006). Third ventricular cavernous Angioma. Med J Malaysia.

[6] Kivelev J, Niemelä M, Kivisaari R, Hernesniemi J (2010). Intraventricular cerebral cavernomas: a series of 12 patients and review of the literature. J Neurosurg.

[7] Patibandla MR, Thotakura AK, Panigrahi MK (2014). Third ventricular cavernous malformation: an unusual lesion. Br J Neurosurg.

[8] Han MS, Moon KS, Lee KH (2014). Cavernous hemangioma of the third ventricle: a case report and review of the literature. World J Surg Oncol.

[9] Beechar VB, Srinivasan VM, Reznik OE (2017). Intraventricular Cavernomas of the third ventricle: report of 2 cases and a systematic review of the literature. World Neurosurg.

[10] Flemming KD (2017). Clinical Management of Cavernous Malformations. Curr Cardiol Rep.

[11] Cox EM, Bambakidis NC, Cohen ML (2017). Pathology of cavernous malformations. Handb Clin Neurol.

[12] Liebelt BD, Boghani Z, Takei H (2015). Epithelioid glioblastoma presenting as massive intracerebral hemorrhage: case report and review of the literature. Surg Neurol Int.

